# Cardio-Oncology’s Modern Approaches to Prevent Doxorubicin-Induced Cardiotoxicity: A Systematic Review

**DOI:** 10.7759/cureus.66215

**Published:** 2024-08-05

**Authors:** Aadi R Palvia, Abhiram Rao Damera, Akshay Rahul Nandi, Shikha Magar, Saloni Patidar, Sachin Kasarla, Vaishnavi Ghantasala, Mishank K Shah, Mayank Goyal

**Affiliations:** 1 Internal Medicine, Kharghar Medicity Hospital, Navi Mumbai, IND; 2 Internal Medicine, MediCiti Institute of Medical Sciences, Hyderabad, IND; 3 Internal Medicine, Dr. B. R. Ambedkar Medical College, Bengaluru, IND; 4 Internal Medicine, Kempegowda Institute of Medical Sciences, Bengaluru, IND; 5 Internal Medicine, Bharati Vidyapeeth Medical College, Pune, IND; 6 Internal Medicine, Gitam Institute of Medical Sciences and Research, Visakhapatnam, IND; 7 Internal Medicine, Apollo Hospitals, Hyderabad, IND; 8 Internal Medicine, Gujarat Medical Education and Research Society (GMERS) Medical College, Vadodara, IND; 9 Internal Medicine, Mayo Clinic, Rochester, USA

**Keywords:** exercise training, dexrazoxane, ace inhibitors and angiotensin receptor blockers, candesartan, anthracycline-induced cardiomyopathy, heart failure, chemotherapy-induced cardiotoxicity, liposomal doxorubicin, conventional doxorubicin

## Abstract

Advances in the field of oncology have led to the advent of doxorubicin (DOX), an anthracycline chemotherapeutic agent, through which cancer survival rates have remarkably improved. There has, however, been a rise in adverse effects from the use of DOX, most notably cardiotoxicity. DOX-induced cardiotoxicity is thought to arise through the generation of reactive oxygen species (ROS), causing mitochondrial dysfunction in the cardiomyocytes. This systematic review followed the Preferred Reporting Items for Systematic Reviews and Meta-Analyses (PRISMA) standards and focused on cancer patients undergoing DOX therapy. The research question addressed interventions aimed at preventing DOX-induced cardiotoxicity. Google Scholar, PubMed, and ScienceDirect databases were used to conduct a systematic search. Next, screening was carried out by reviewing the title and abstract of various articles to exclude irrelevant studies, followed by the retrieval of full-text articles. Scale for the assessment of narrative review articles 2 (SANRA 2) for narrative reviews, a measurement tool to assess systematic reviews (AMSTAR) checklist for systematic reviews, and the Cochrane risk of bias tool for randomized controlled trials (RCTs) were the tools employed for quality assessment. This systematic review provides convincing evidence about preventive interventions to counteract DOX-induced cardiotoxicity. Primary prevention strategies against DOX-induced cardiotoxicity include pharmacological and non-pharmacological measures. Dexrazoxane reduces cardiotoxicity without therapeutic compromise. Beta-blockers showed mixed results in preserving cardiac function. The research on renin-angiotensin-aldosterone system (RAAS) inhibitors suggests that most of these agents can reduce the risk of DOX-induced cardiotoxicity. The liposomal formulation of DOX decreases cardiotoxicity without sacrificing effectiveness. Chemotherapy regimens should be supplemented with cardioprotective medications to increase therapeutic efficacy and lower cardiac risks. Exercise is an essential non-pharmacological strategy for decreasing DOX-induced cardiotoxicity. It acts by lowering oxidative stress, maintaining mitochondrial function, and averting apoptosis. Other non-pharmacological interventions through antioxidative, anti-apoptotic, and mitochondrial protective mechanisms, such as resveratrol, vitamin E, curcumin, and visnagin, show promise in lowering DOX-induced cardiotoxicity and may be useful as supplementary therapy during cancer treatment. In conclusion, this review highlights the need for a multimodal strategy that incorporates different tactics, as well as the need for additional research and strong clinical trials, with the ultimate goal of protecting cardiac health in patients receiving chemotherapy with DOX.

## Introduction and background

Doxorubicin (DOX) is an anthracycline chemotherapeutic agent used to treat various types of malignancies, especially breast cancer and various leukemias. It stops the malignant cells from proliferating and activates apoptosis in them by intercalating deoxyribonucleic acid (DNA). It is one of our strongest weapons against cancer, but unfortunately, it is highly cardiotoxic. Its adverse effects on the circulatory system cause a dual problem. First, it limits the clinical utility while treating cancer patients, and then it complicates and severely deteriorates the prognosis, which was bad in the first place, by damaging the heart. Survival rates have improved remarkably as a result of advances in the field of oncology, but these advancements have also been followed by a rise in adverse effects associated with chemotherapy, most notably DOX-induced cardiotoxicity. It presents with a range of cardiovascular complications, including heart failure (HF), arrhythmias, cardiomyopathy, and death [1]. HF caused by DOX usually carries a worse prognosis compared to other HF causes, especially in those cases with a delayed diagnosis. Up to 50% of patients die within two years of diagnosis. The pathology of DOX-induced cardiotoxicity is intricate and poorly understood. DOX-induced cardiotoxicity is caused by reactive oxygen species (ROS) and mitochondrial dysfunction. As DOX builds up in cardiac mitochondria, it inhibits Topoisomerase 2β (TOP2B), causing mitochondrial malfunction and the production of ROS. This encourages remodeling of the heart and apoptosis, two processes crucial to the development of DOX-induced cardiotoxicity [2]. There are major genetic factors involved, including the mutation in the retinoic acid receptor gamma (RARG) S427L gene. Altering the expression of RARG and subsequently TOP2B, which is associated with the mechanism of DOX's cardiotoxic effects, results in increased vulnerability to DOX-induced cardiotoxicity [1].

The use of dexrazoxane and beta-blockers are examples of preventive measures. Dexrazoxane has cardioprotective properties, but it can also cause myelosuppression and possibly reduce the efficacy of DOX. The potency of carvedilol, a beta-blocker with ROS-suppressive qualities, in reducing DOX-induced cardiotoxicity has been inconsistent [3]. Using renin-angiotensin-aldosterone system inhibitors (RAAS), such as angiotensin receptor blockers (ARBs) and angiotensin-converting enzyme (ACE) inhibitors, is also recommended. These medications may be able to preserve heart function by decreasing oxidative stress and, thereby, halting myocardial remodeling. In spite of progress, a number of obstacles still exist. The lack of predictive indicators for identifying people who are more likely to experience cardiotoxicity makes it challenging to customize prophylactic strategies [4]. The use of ACE inhibitors, ARBs, and beta-blockers is not generally recognized due to concerns about potential adverse effects and their effectiveness in reducing cardiotoxicity in various patient populations [5]. DOX-induced cardiotoxicity is dose-dependent, yet there is no clear cut-off threshold for starting cardioprotective measures [6]. Although mostly asymptomatic, most DOX-induced cardiotoxicity occurs during the first year after therapy, necessitating early identification for better patient outcomes [4]. Traditional parameters are frequently employed for the diagnosis of DOX-induced cardiotoxicity, such as a reduction in left ventricular ejection fraction (LVEF). However, these metrics do not fully account for the spectrum of cardiotoxicity, which may also include arrhythmias and increased cardiac biomarkers. Echocardiograms (ECHO) and cardiac magnetic resonance imaging can be used to identify subclinical cardiotoxicity. Monitoring cardiac damage can also benefit from the use of biomarkers such as high-sensitivity cardiac troponin T and NT-proB-type natriuretic peptides. These diagnostic methods can be used to guide early intervention, enabling timely alterations to chemotherapy regimens, which can stop DOX-induced cardiotoxicity from progressing. Focused research on innovative techniques, such as non-ischemic pharmacologic preconditioning with docosahexaenoic acid (DHA) and dual antioxidant strategies that combine DHA and carvedilol, aims to reduce oxidative stress, which is an essential factor in reducing the onset and progression of DOX-induced cardiotoxicity [3]. Targeted treatments such as liposomal forms of DOX are being developed to reduce cardiotoxicity without sacrificing chemotherapeutic activity [5]. When compared to traditional anthracyclines, liposomal doxorubicin (L-DOX) lessens cardiotoxicity significantly by decreasing its exposure in normal tissues while retaining therapeutic efficacy in cancer cells [6]. New biomarkers such as myeloperoxidase and interleukin-6 are also being explored for the identification and early prediction of cardiotoxicity [7].

## Review

Methods

The Preferred Reporting Items for Systematic Reviews and Meta-Analyses (PRISMA) standards were followed for this systematic review. In accordance with the participants, intervention, and outcome (PIO) components, the following research question was developed: participants in the study included cancer patients receiving DOX therapy; the intervention was centered on pharmacological strategies to prevent cardiotoxicity; and the outcomes of interest included cardiac function, survival rates, and improvements in quality of life. The inclusion criteria specified that articles had to be in English, available as free full-text, and published within the last decade. Exclusion criteria included papers published in languages other than English, those unrelated to the research topic, and those without full-text accessibility.

A systematic search of the major search engines and databases was performed, utilizing Google Scholar, PubMed, and ScienceDirect databases. All of the datasets were last searched in July of 2024. DOX, cardiotoxicity, therapeutic methods, and the medical subject heading (MeSH) technique employed by PubMed were the search engine keywords. Table 1 contains specifics about the databases and search techniques.

**Table 1:** Detailed description of databases' search terms and results

Databases	Search strategy	Number of articles before filters	Filters	Search result
PubMed	Cardiotoxicity OR ( "Cardiotoxicity/diagnosis"[Majr] OR "Cardiotoxicity/diet therapy"[Majr] OR "Cardiotoxicity/drug therapy"[Majr] OR "Cardiotoxicity/etiology"[Majr] OR "Cardiotoxicity/mortality"[Majr] OR "Cardiotoxicity/pathology"[Majr] OR "Cardiotoxicity/physiopathology"[Majr] OR "Cardiotoxicity/prevention and control"[Majr] OR "Cardiotoxicity/therapy"[Majr] ) AND Doxorubicin OR ( "Doxorubicin/administration and dosage"[Majr] OR "Doxorubicin/adverse effects"[Majr] OR "Doxorubicin/antagonists and inhibitors"[Majr] OR "Doxorubicin/poisoning"[Majr] OR "Doxorubicin/therapeutic use"[Majr] OR "Doxorubicin/toxicity"[Majr] )	16,940	Free full text, from 2014 to 2024, 10 years, Humans, English	1450
Google Scholar	Cardiotoxicity AND Doxorubicin	1430	2014-2024	906
Science Direct	Cardiotoxicity AND Doxorubicin	15,015	2014-2024, English, open access and open archive	2,227

All the records were screened based on the titles and abstracts, and the exclusion of irrelevant studies was implemented. Retrieval of the full-text articles was done. The articles successfully retrieved were assessed according to the appropriate tool for quality appraisal to minimize the risk of bias in this study. 

The quality and bias of the retrieved full-text articles were assessed using specific tools designed for each type of study: the Cochrane risk of bias tool was used for randomized controlled trials (RCT), a measurement tool to assess systematic reviews (AMSTAR) checklist was used for systematic reviews, and the scale for the assessment of narrative review articles 2 (SANRA 2) was used for narrative reviews [8-10]. Each tool has its own set of requirements and minimum scores needed to pass.

Results

**Figure 1:**
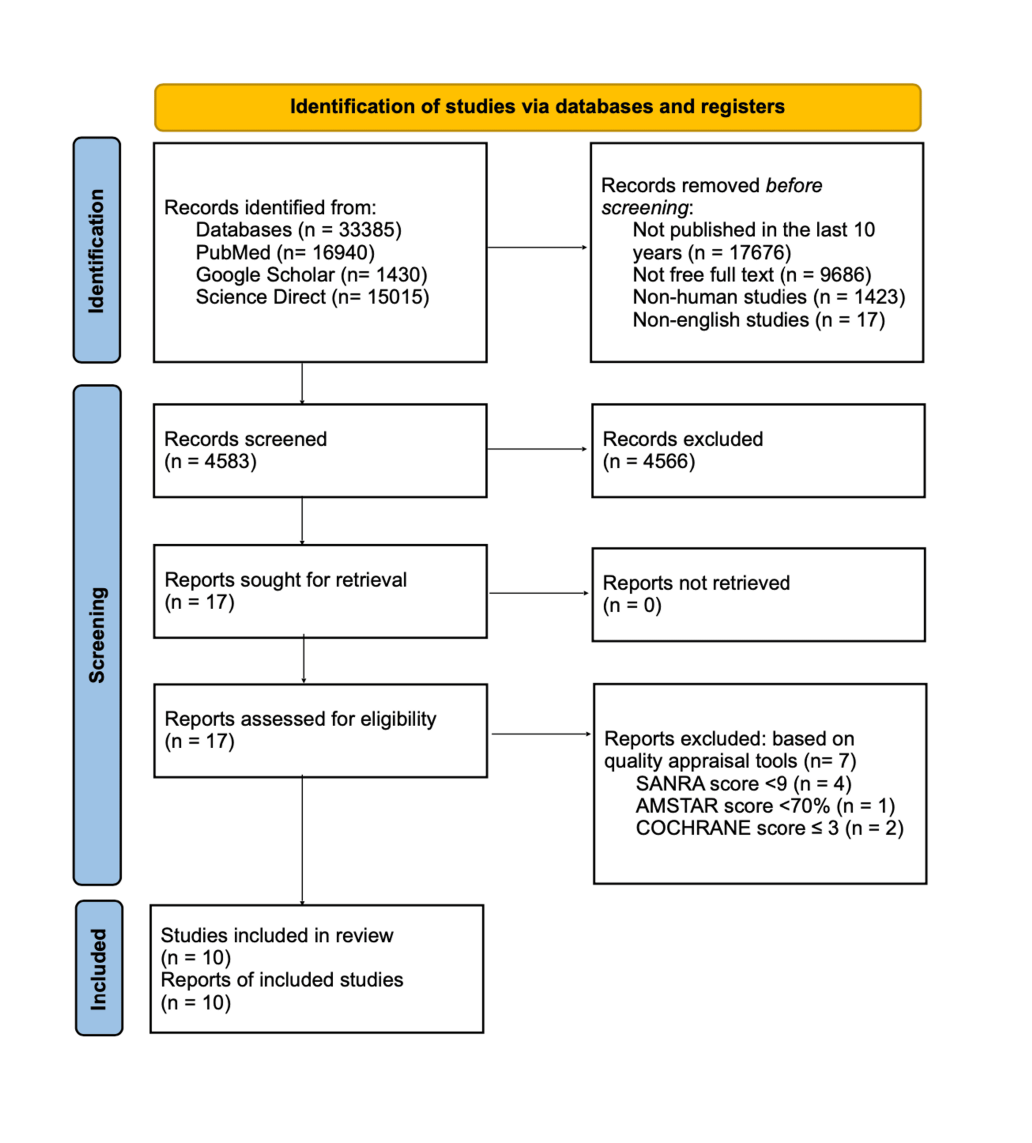
PRISMA chart PRISMA: Preferred Reporting Items for Systematic Reviews and Meta-Analyses

The database search started with 33,385 potentially relevant titles. 4,583 records remained after applying the inclusion and exclusion criteria. In the following stage, 4,565 publications were excluded while checking the records' titles and abstracts against this review's PIO components and eligibility criteria, leaving 17 papers for retrieval. A total of 17 papers were included for a thorough quality/bias assessment using standardized quality assessment procedures. After a quality analysis, 10 research studies were included, and seven papers were found to be unsuitable for inclusion in this systematic review. Figure 1 presents a flow diagram that illustrates the screening procedure and study selection.

Table 2 provides information regarding the characteristics of the selected studies.

**Table 2:** Characteristics of the selected studies after screening ACE, angiotensin-converting enzyme; ARB, angiotensin receptor blocker; RNA, ribonucleic acid; PDE-5, phosphodiesterase-5; L-DOX, liposomal doxorubicin; RCT, randomized control trial

Author	Year	Type of study	Preventive intervention
Neha Bansal, et al. [11]	2019	Narrative review	Dexrazoxane, beta-blockers, ACE inhibitors, ARB, aldosterone antagonists, statins, and exercise
Pimprapa Vejpongsa, et al. [12]	2014	Narrative review	Dexrazoxane, L-DOX, beta-blockers, ACE inhibitors, and ARB
Kelly Liesse, et al. [13]	2018	Systematic review	Dexrazoxane
Asdi Wihandono, et al. [14]	2021	RCT	Lisinopril and bisoprolol
Paweł Sobczuk, et al. [15]	2022	Narrative review	ACE inhibitors, ARB, and aldosterone antagonists
Myunhee Lee, et al. [16]	2021	RCT	Candesartan and carvedilol
Franco YL, et al. [17]	2018	Narrative review	L-DOX
Bin Bin Wu, et al. [18]	2022	Narrative review	Mitochondrial transplantation, microRNAs, and human pluripotent stem cell
Pushkar Singh Rawat, et al. [19]	2021	Narrative review	Metformin, statins, beta-blockers, ACE inhibitors, PDE-5 inhibitors, alpha 1-adrenergic agonists, dexrazoxane, resveratrol, vitamin E, probucol, allicin, erythropoietin, curcumin, visnagin, and schisandrin B
Sanela Dozic, et al. [20]	2023	Narrative review	Exercise

Tables 3-5 show the quality/bias assessment's outcome as well as how each study was assessed in relation to its relevant study category. Table 3 shows the assessment of narrative reviews using the SANRA 2 checklist. Table 4 demonstrates the AMSTAR checklist for systematic review in this review. Finally, Table 5 demonstrates the assessment process of RCT using the Cochrane risk of bias tool.

**Table 3:** Results of the SANRA 2 assessment tool for narrative reviews Passing score is 9/12 [8]. SANRA, a scale for the quality assessment of narrative review articles

First author, year	Justification of the article’s importance for the readership	Statement of concrete aims or formulation of the question	Description of the literature search	Referencing	Scientific reasoning	Appropriate presentation of data	Sum	Pass/fail
Neha Bansal, et al., 2019 [11]	2	2	0	2	2	2	10	Pass
Pimprapa Vejpongsa, et al., 2014 [12]	2	1	0	2	2	2	9	Pass
Paweł Sobczuk, et al., 2022 [15]	2	2	0	2	2	2	10	Pass
Yesenia L Franco, et al., 2018 [17]	2	2	1	2	2	2	11	Pass
Bin Bin Wu, et al., 2022 [18]	1	2	0	2	2	2	9	Pass
Pushkar Singh Rawat, et al., 2021 [19]	2	2	0	2	2	2	10	Pass
Sanela Dozic, et al., 2023 [20]	2	2	0	2	1	2	9	Pass
Pierantonio Menna, et al., 2017 [21]	1	1	0	1	2	2	7	Fail
Izabela Koss-Mikołajczyk, et al., 2021 [22]	1	2	0	2	1	1	7	Fail
Jing Zhang, et al., 2016 [23]	2	2	0	1	2	1	8	Fail
Konrad Teodor Sawicki, et al., 2021 [24]	2	1	0	2	1	2	8	Fail

**Table 4:** Details of AMSTAR checklist for systematic review articles retained in our study To check yes, no, can’t answer, and not applicable. The passing score is >70% [9]. AMSTAR, a measurement tool to assess systematic reviews

Checklist	Kelly Liesse, et al., 2019 [13]	Hany Akeel Al-hussaniy, et al., 2023 [25]
Was an "a priori" design provided?	Yes	Yes
Were there duplicates in study selection and data extraction?	No	Yes
Was a comprehensive literature search performed?	Yes	Yes
Was the status of publication (e.g., grey literature) used as an inclusion criterion?	Yes	Yes
Was a list of studies (included and excluded) provided?	No	No
Were the characteristics of the included studies provided?	Yes	No
Was the scientific quality of the included study used appropriately in formulating conclusions?	Yes	Yes
Were the methods used to combine the findings of studies appropriate?	Yes	No
Was the likelihood of publication bias assessed?	Yes	No
Was the conflict of interest included?	Yes	Yes
Total score	80%	60%
Pass/fail	Pass	Fail

**Table 5:** Critical appraisal using the Cochrane risk of bias tool for RCT Passing score is 50% [10]. RCT, randomized control trial

Checklist	Selection bias (random sequence generation)	Selection bias (allocation concealment)	Reporting bias	Performance bias	Detection bias	Attrition bias	Overall quality low on risk of bias	Other bias	Pass/fail
Asdi Wihandono, et al., 2021 [14]	Low risk of bias	Unclear risk of bias	Low risk of bias	Low risk of bias	Low risk of bias	Low risk of bias	5/6	None	Pass
Myunhee Lee, et al., 2021 [16]	Low risk of bias	Low risk of bias	Low risk of bias	Unclear risk of bias	Unclear risk of bias	Low risk of bias	4/6	None	Pass
Peter Georgakopoulos, et al., 2019 [26]	Low risk of bias	High risk of bias	Unclear risk of bias	Low risk of bias	Low risk of bias	Low risk of bias	3/6	None	Fail
Shinjeong Song, et al., 2024 [27]	Low risk of bias	Unclear risk of bias	Unclear risk of bias	Unclear risk of bias	Unclear risk of bias	Unclear risk of bias	1/6	None	Fail

Discussion

Current Therapeutic Approaches and Their Efficacy

Primary prevention strategies to prevent DOX-induced cardiotoxicity are dexrazoxane and neurohormonal blocking drugs. Dexrazoxane is a drug that provides cardioprotection by binding iron and preventing the formation of free radicals, which lead to cardiac damage [11]. It has been shown to be effective in preventing cardiomyopathy without affecting the treatment outcomes of individuals with breast cancer and leukemia. Despite this, there are concerns about its impact on secondary malignancies [12]. The study by Kelly Liesse et al. examined the effects of different dosages of anthracyclines and the benefit of dexrazoxane on cardiac health. Increased doses of anthracyclines in the absence of dexrazoxane result in an increase in DOX-induced cardiotoxicity, although dexrazoxane helps delay these cardiac issues. Nevertheless, the use of dexrazoxane only marginally increases overall survival chances [13].

Beta-blockers (such as carvedilol and metoprolol) are also being studied for their antioxidant qualities and potential to reduce cardiotoxicity by inhibiting pathways that contribute to the progression of cardiovascular disease. When used in conjunction with anthracycline therapy, beta-blockers have been demonstrated to have variable effects on LVEF preservation and the reduction of cardiac biomarkers such as serum cardiac troponin I concentrations [12].

Research by Wihandono et al. examined the effects of lisinopril and bisoprolol on decreasing DOX-induced cardiotoxicity in patients with locally advanced breast cancer receiving neoadjuvant chemotherapy. 51 women were enlisted in this trial and randomized to receive chemotherapy with either lisinopril and bisoprolol or chemotherapy alone. LVEF was assessed by ECHO both prior to and following the sixth treatment round. The treatment of lisinopril along with bisoprolol resulted in substantial preservation of cardiac function, as shown by the mean change in LVEF. The study found that the combined use of bisoprolol and lisinopril during chemotherapy may be able to reduce DOX-induced cardiotoxicity. However, there is a need for longer follow-ups in order to evaluate long-term cardiac outcomes [14].

RAAS alteration is one of the multifactorial aspects of DOX-induced cardiotoxicity. DOX promotes inflammation, fibrosis, and apoptosis in cardiac tissues. ACE inhibitors and ARBs are two examples of RAAS inhibitors that have shown substantial potential in the prevention and treatment of DOX-induced cardiotoxicity. For high-risk patients on anthracycline therapy, the latest recommendations suggest using RAAS inhibitors, beta-blockers, and dexrazoxane [15]. ACE inhibitors and ARBs such as enalapril and valsartan have shown some promise in reducing LVEF decline and cardiac biomarkers, but definitive recommendations require additional research, particularly on long-term adverse effects such as HF. The preventive actions of zofenopril, a newer ACE inhibitor, are based on unique signaling pathways regulating cell survival via hydrogen sulfide, which is derived from the sulfhydryl group, in addition to ACE inhibition. It has been demonstrated in many cardiovascular diseases that hydrogen sulfide donors produce vasodilation, induce anti-inflammatory responses, and protect the cardiac tissue [11,15]. Statins may have a cardioprotective effect when used in conjunction with anthracycline therapy; however, more research is needed to determine their effectiveness and safety [11]. Secondary preventive measures focus on biomarkers such as troponin and ECHO for early detection, which allows for prompt pharmaceutical management to decrease cardiac dysfunction [12].

The study by Myunhee Lee et al. aimed to ascertain if low-dose candesartan might effectively lower DOX-induced cardiotoxicity in female breast cancer patients who had not previously had cardiovascular risk factors following DOX treatment. The trial consisted of 195 individuals who were randomly assigned to receive candesartan, carvedilol, or no medication (control). It was shown that candesartan, in contrast to carvedilol and the control, significantly reduced the incidence of early DOX-induced cardiotoxicity. The outcomes showed that, in comparison to the control group, carvedilol had some protective effects against early DOX-induced cardiotoxicity, although not as much as candesartan. Furthermore, candesartan maintained a higher LVEF and reduced cardiotoxicity up to one year after chemotherapy, demonstrating long-lasting preventive results. These findings suggest that, even though carvedilol may have some short-term cardioprotective effects, candesartan appears to be more effective than carvedilol in preventing and treating DOX-induced cardiotoxicity [16].

**Figure 2:**
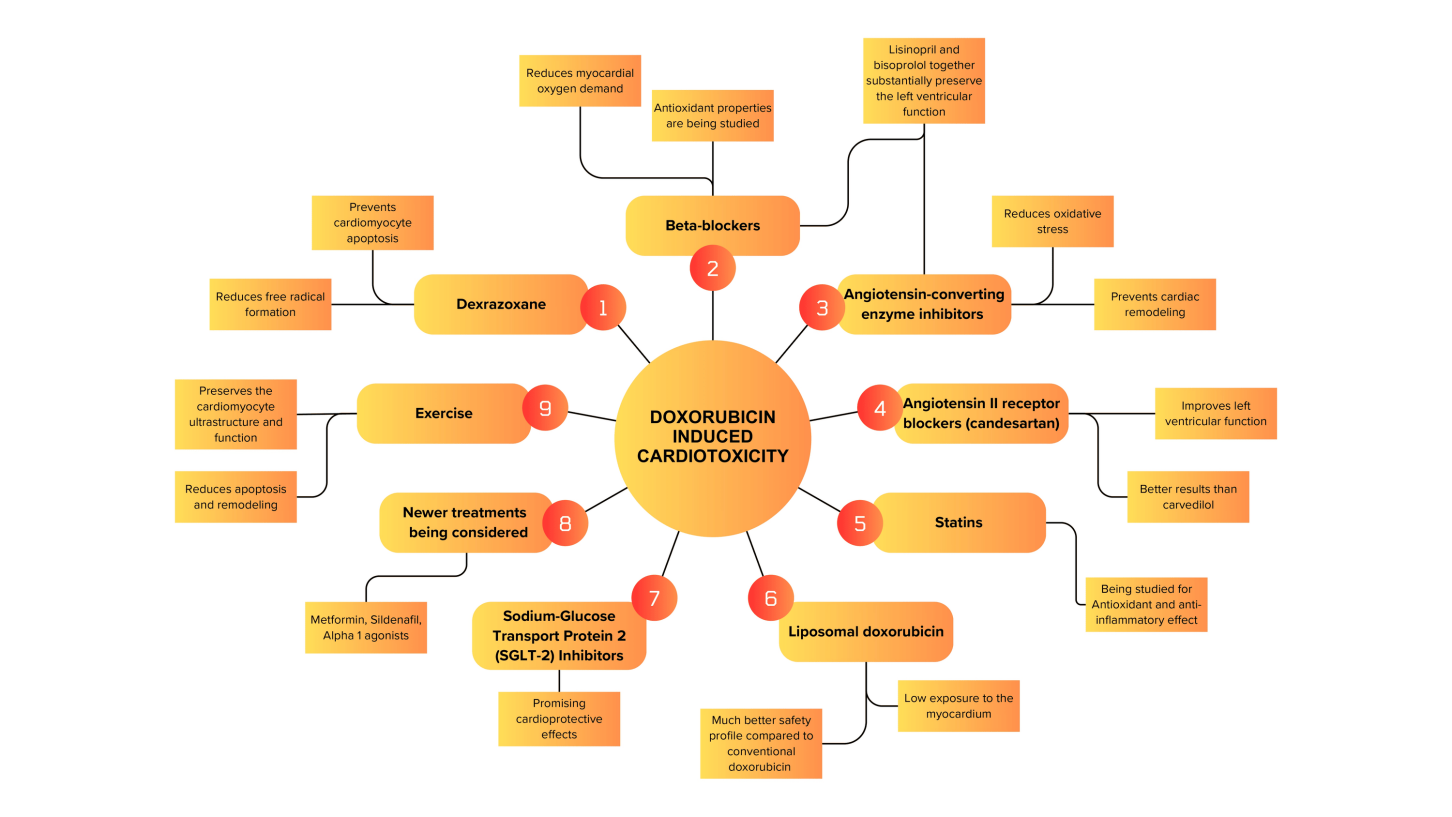
Various preventive strategies to counteract DOX-induced cardiotoxicity DOX, doxorubicin Created by Vaishnavi Ghantasala and Mishank K. Shah.

The use of L-DOX is a fascinating approach to preventing the toxic effects of conventional DOX while retaining its therapeutic potency, as this tailored administration reduces systemic exposure and damage to healthy cardiac tissue. This is particularly helpful for patients with pre-existing cardiac ailments and patients on concurrent trastuzumab therapy. Phase II and III clinical trials have well established that L-DOX reduces the incidence of cardiotoxicity when compared to conventional DOX. This validates the safety and efficacy of L-DOX in the treatment of cancer and promotes its use in a wider clinical setting [17]. Figure 2 shows the various preventive strategies along with their effects.

Table 6 shows a few preventive strategies against DOX-induced cardiotoxicity in a comparative manner.

**Table 6:** Comparative analysis of preventive pharmacological interventions ACE, angiotensin-converting enzyme; ARB, angiotensin II receptor blocker; LVEF, left ventricular ejection fraction; DOX: doxorubicin; L-DOX, liposomal doxorubicin

Intervention	Mechanism	Effectiveness	Studies/findings	Concerns/limitations
Dexrazoxane	It binds to iron and inhibits free radical formation.	Effective in reducing clinical and subclinical cardiotoxicity.	Consistent efficacy in preventing cardiomyopathy, especially in breast cancer and childhood leukemia.	Potential impact on secondary malignancies.
ACE inhibitors	Inhibits ACE and reduces angiotensin II levels.	Significant efficacy in preventing and treating DOX-induced cardiotoxicity.	Enalapril has been shown to mitigate LVEF decline.	Further research is needed for definitive recommendations.
ARB	Inhibits the angiotensin II receptor.	Effective in reducing early DOX-induced cardiotoxicity occurrences and maintaining higher LVEF.	A study showed that candesartan significantly reduced DOX-induced cardiotoxicity compared to carvedilol.	Further research is needed for long-term clinical outcomes.
Beta-blockers	Antioxidant properties and blocking pathways contributing to heart disease progression.	Mixed results in preserving LVEF and reducing cardiac biomarkers.	Studies show mixed results on LVEF preservation and a reduction in serum cardiac troponin I concentrations.	Varied effectiveness, need for more robust research.
L-DOX	Targeted delivery to minimize systemic exposure and toxicity.	Lower incidences of cardiotoxicity while maintaining therapeutic effectiveness.	Phase II and III studies show lower cardiotoxicity incidences compared to conventional DOX.	Further research is needed for broader adoption in clinical practice.
Statins	Potential cardioprotective properties.	Efficacy and safety are under ongoing study.	Research on their role during anthracycline treatment is being debated.	Unclear efficacy and safety, especially in pediatric populations.

Novel and Emerging Therapies

The mitochondria play a key role in DOX-induced cardiotoxicity by changing energy production, generating ROS, and activating cell death pathways. Altering the way that the drugs are administered, such as switching from bolus injection to continuous infusion, reduces the peak plasma concentration and potentially minimizes the amount of DOX being exposed to the cardiac tissue. The objective of antioxidant therapies like alpha-tocopherol and N-acetylcysteine is to battle oxidative stress [18]. In preclinical research, novel strategies like mitochondrial transplantation and targeted mitochondrial division inhibitor-1 have the potential to preserve mitochondrial function and lower cardiomyocyte apoptosis in response to DOX. The reflection of these innovative preclinical discoveries into pragmatic real-life clinical treatments continues to pose a challenge. Robust confirmation is required by clinical trials [19]. Using current therapeutics against HF medications like sodium-glucose cotransporter-2 (SGLT2) inhibitors also shows great promise in protecting the cardiac tissue against the insults of DOX. Human pluripotent stem cell-derived cardiomyocytes are of utmost importance as they allow for the study of DOX-induced cardiotoxicity in a human-relevant model. These cells allow us to gain valuable insights into the disease mechanics and provide us with newer therapeutic targets like microRNAs. However, achieving mature and functional cardiomyocytes still remains an obstacle, which limits its utility in drug screening and personalized applications [18]. Metformin activates adenosine monophosphate-activated protein kinase (AMPK) signaling inhibition and activation of the apoptotic pathways, reducing oxidative stress and preserving cardiac function. Statins like pitavastatin shield against DOX-induced cardiotoxicity through RNS/Rac1-dependent AMPK pathway modulation. Phosphodiesterase-5 (PDE-5) inhibitors such as sildenafil conserve mitochondrial integrity and counteract ventricular dysfunction [19]. Alpha-1 adrenergic agonists reduce apoptotic and fibrotic activity in cardiac muscle, thereby conserving mitochondrial function. MiR-181c, a microRNA, averts cardiomyocyte apoptosis via the PI3K/Akt pathway, providing defense against DOX-induced cardiotoxicity. Drugs such as allicin, probucol, and erythropoietin enhance antioxidant defenses and decrease cardiac injury [19].

Non-Pharmacological Interventions and Lifestyle Modifications

Exercise is an important preventative intervention against DOX-induced cardiotoxicity, as suggested strongly by preclinical and clinical studies. It has proven to decrease oxidative stress by enhancing endogenous antioxidants and, hence, conserving mitochondrial function, which is critical for preventing mitochondrial-mediated apoptosis. Exercise upregulates heat shock proteins, preserves cardiomyocyte ultrastructure, and reduces fibrosis, thereby preserving cardiac function. It conserves cardiac size and function, improves vascular health, and, in general, is cardioprotective against the insults of DOX [20]. Many natural compounds demonstrate the potential for averting DOX-induced cardiotoxicity via various mechanisms. Resveratrol activates AMPK to decrease oxidative stress, apoptosis, and fibrosis. Vitamin E is an antioxidant that protects cell membranes from ROS. Ginsenoside Rh2 improves cell survival and decreases cardiomyocyte injury. Curcumin reduces cardiomyocyte pyroptosis, decreases ROS levels, and activates the PI3K/Akt/mTOR pathway. Visnagin hampers the activity of mitochondrial malate dehydrogenase, hence preserving mitochondrial function. Schisandrin B potentiates cardiomyocyte glutathione redox cycling and reverses DOX-induced effects on cardiac tissue. These compounds represent promising adjunctive therapies targeting oxidative stress, apoptosis, and mitochondrial dysfunction during cancer treatment [19].

Limitations

Despite the comprehensive overview of current therapeutic approaches and their efficacy in preventing DOX-induced cardiotoxicity, several limitations must be acknowledged. The literature used to create this review may differ significantly in terms of design, sample size, patient characteristics, and treatment regimen, which might make it a challenge to draw a definitive conclusion and generalize findings for a diverse patient population on a global scale. Some therapies may have the potential to cause significant adverse reactions and might be a cause for concern. A more detailed risk-benefit analysis is needed in terms of large-scale clinical trials. Finally, the therapeutic strategies for the prevention of DOX-induced cardiotoxicity are subject to change over time, and newer studies will be needed in the future.

## Conclusions

DOX causes serious cardiotoxicity, which may lead to significant mortality and morbidity in patients undergoing chemotherapy. A robust preventive strategy is crucial in order to improve patient outcomes. Dexrazoxane is one such approach that can be implemented both in clinical and subclinical cardiotoxicity without compromising the positive effects of DOX. Beta-blockers are being researched for their antioxidant properties. RAAS blockers like ACE inhibitors and ARBs show promise for reducing the rate of decline in LVEF, demonstrating their effectiveness in preventing and potentially treating DOX-induced cardiotoxicity. It is hypothesized that this effect is achieved by blocking the apoptotic, fibrotic, and inflammatory pathways. L-DOX offers a reduction in cardiotoxicity over conventional DOX due to its improved pharmacodynamic properties. Significant advances can be achieved by using repurposed HF drugs (e.g., SGLT2 inhibitors), human pluripotent stem cell-derived cardiomyocytes, and therapeutic targets like microRNAs to understand the processes leading to DOX-induced cardiotoxicity. A non-pharmacological strategy for mitigating DOX-induced cardiotoxicity is exercise. It goes a long way toward lowering oxidative stress and preserving mitochondrial function while promoting heart and vascular health. In conclusion, a multimodal strategy integrating pharmaceutical therapies and lifestyle changes is necessary for the prevention of DOX-induced cardiotoxicity. Further research and clinical trials that verify these strategies and optimize treatment procedures are required to achieve the goal of improving patient outcomes and quality of life both during and after cancer therapy.

## References

[1] Carrasco R, Ramirez MC, Nes K (2020). Prevention of doxorubicin-induced Cardiotoxicity by pharmacological non-hypoxic myocardial preconditioning based on Docosahexaenoic Acid (DHA) and carvedilol direct antioxidant effects: study protocol for a pilot, randomized, double-blind, controlled trial (CarDHA trial). Trials.

[2] Carrasco R, Castillo RL, Gormaz JG, Carrillo M, Thavendiranathan P (2021). Role of oxidative stress in the mechanisms of anthracycline-induced cardiotoxicity: effects of preventive strategies. Oxid Med Cell Longev.

[3] Volkova M, Russell R 3rd (2011). Anthracycline cardiotoxicity: prevalence, pathogenesis and treatment. Curr Cardiol Rev.

[4] Neves MF (2023). Renin-angiotensin system inhibition and beta blockade adrenergic may be useful to attenuate cardiotoxicity by anthracyclines. Arq Bras Cardiol.

[5] Silva EN, Ribeiro ML, Caldeira LC (2024). Biomarkers and prediction of anthracyclic cardiotoxicity in breast cancer. Rev Assoc Med Bras (1992).

[6] Zhang J, Jiang H, Zhang J, Bao G, Zhang G, Wang H, Wang X (2021). Effectiveness and safety of pegylated liposomal doxorubicin versus epirubicin as neoadjuvant or adjuvant chemotherapy for breast cancer: a real-world study. BMC Cancer.

[7] Kettana KM, El-Haggar SM, Alm El-Din MA, El-Afify DR (2024). Possible protective effect of rosuvastatin in chemotherapy-induced cardiotoxicity in HER2 positive breast cancer patients: a randomized controlled trial. Med Oncol.

[8] Baethge C, Goldbeck-Wood S, Mertens S (2019). SANRA-a scale for the quality assessment of narrative review articles. Res Integr Peer Rev.

[9] Shea BJ, Reeves BC, Wells G (2017). AMSTAR 2: a critical appraisal tool for systematic reviews that include randomised or non-randomised studies of healthcare interventions, or both. BMJ.

[10] Higgins JP, Altman DG, Gøtzsche PC (2011). The Cochrane collaboration's tool for assessing risk of bias in randomised trials. BMJ.

[11] Bansal N, Adams MJ, Ganatra S (2019). Strategies to prevent anthracycline-induced cardiotoxicity in cancer survivors. Cardiooncology.

[12] Vejpongsa P, Yeh ET (2014). Prevention of anthracycline-induced cardiotoxicity: challenges and opportunities. J Am Coll Cardiol.

[13] Liesse K, Harris J, Chan M, Schmidt ML, Chiu B (2018). Dexrazoxane significantly reduces anthracycline-induced cardiotoxicity in pediatric solid tumor patients: a systematic review. J Pediatr Hematol Oncol.

[14] Wihandono A, Azhar Y, Abdurahman M, Hidayat S (2021). The role of lisinopril and Bisoprolol to prevent anthracycline induced cardiotoxicity in locally advanced breast cancer patients. Asian Pac J Cancer Prev.

[15] Sobczuk P, Czerwińska M, Kleibert M, Cudnoch-Jędrzejewska A (2022). Anthracycline-induced cardiotoxicity and renin-angiotensin-aldosterone system-from molecular mechanisms to therapeutic applications. Heart Fail Rev.

[16] Lee M, Chung WB, Lee JE, Park CS, Park WC, Song BJ, Youn HJ (2021). Candesartan and carvedilol for primary prevention of subclinical cardiotoxicity in breast cancer patients without a cardiovascular risk treated with doxorubicin. Cancer Med.

[17] Franco YL, Vaidya TR, Ait-Oudhia S (2018). Anticancer and cardio-protective effects of liposomal doxorubicin in the treatment of breast cancer. Breast Cancer (Dove Med Press).

[18] Wu BB, Leung KT, Poon EN (2022). Mitochondrial-targeted therapy for doxorubicin-induced cardiotoxicity. Int J Mol Sci.

[19] Rawat PS, Jaiswal A, Khurana A, Bhatti JS, Navik U (2021). Doxorubicin-induced cardiotoxicity: an update on the molecular mechanism and novel therapeutic strategies for effective management. Biomed Pharmacother.

[20] Dozic S, Howden EJ, Bell JR, Mellor KM, Delbridge LM, Weeks KL (2023). Cellular mechanisms mediating exercise-induced protection against cardiotoxic anthracycline cancer therapy. Cells.

[21] Menna P, Salvatorelli E (2017). Primary prevention strategies for anthracycline cardiotoxicity: a brief overview. Chemotherapy.

[22] Koss-Mikołajczyk I, Todorovic V, Sobajic S, Mahajna J, Gerić M, Tur JA, Bartoszek A (2021). Natural products counteracting cardiotoxicity during cancer chemotherapy: the special case of doxorubicin, a comprehensive review. Int J Mol Sci.

[23] Zhang J, Cui X, Yan Y, Li M, Yang Y, Wang J, Zhang J (2016). Research progress of cardioprotective agents for prevention of anthracycline cardiotoxicity. Am J Transl Res.

[24] Sawicki KT, Sala V, Prever L, Hirsch E, Ardehali H, Ghigo A (2021). Preventing and treating anthracycline cardiotoxicity: new insights. Annu Rev Pharmacol Toxicol.

[25] Al-Hussaniy HA, Alburghaif AH, Alkhafaje Z (2023). Chemotherapy-induced cardiotoxicity: a new perspective on the role of Digoxin, ATG7 activators, Resveratrol, and herbal drugs. J Med Life.

[26] Georgakopoulos P, Kyriakidis M, Perpinia A (2019). The role of metoprolol and enalapril in the prevention of doxorubicin-induced cardiotoxicity in lymphoma patients. Anticancer Res.

[27] Song S, Woo J, Kim H, Lee JW, Lim W, Moon BI, Kwon K (2024). A prospective randomized controlled trial to determine the safety and efficacy of extracorporeal shock waves therapy for primary prevention of subclinical cardiotoxicity in breast cancer patients without a cardiovascular risk treated with doxorubicin. Front Cardiovasc Med.

